# Severe Fatigue and Memory Impairment Are Associated with Lower Serum Level of Anti-SARS-CoV-2 Antibodies in Patients with Post-COVID Symptoms

**DOI:** 10.3390/jcm10194337

**Published:** 2021-09-23

**Authors:** Tihamer Molnar, Reka Varnai, Daniel Schranz, Laszlo Zavori, Zoltan Peterfi, David Sipos, Margit Tőkés-Füzesi, Zsolt Illes, Andras Buki, Peter Csecsei

**Affiliations:** 1 Department of Anaesthesiology and Intensive Care, University of Pecs, Medical School, H7632 Pecs, Hungary; molnar.tihamer@pte.hu; 2 Department of Primary Health Care, University of Pecs, Medical School, H7632 Pecs, Hungary; 3 Department of Neurology, University of Pecs, Medical School, H7632 Pecs, Hungary; schranzdan@gmail.com; 4 Salisbury NHS Foundation Trust, Salisbury SP2 8BJ, UK; zavori.laszlo@gmail.com; 5 1st Department of Internal Medicine, Division of Infectology, University of Pecs, Medical School, H7632 Pecs, Hungary; peterfi.zoltan@pte.hu (Z.P.); sipos.david@pte.hu (D.S.); 6 Department of Laboratory Medicine, University of Pecs, Medical School, H7632 Pecs, Hungary; tfgitta@gmail.com; 7 Department of Neurology, Odense University Hospital, University of Southern Denmark, 5230 Odense, Denmark; zsolt.illes@rsyd.dk; 8 Department of Neurosurgery, University of Pecs, Medical School, H7632 Pecs, Hungary; buki.andras@pte.hu (A.B.); csecsei.peter@pte.hu (P.C.)

**Keywords:** post-COVID fatigue, anti-SARS-CoV-2 spike Ig, anti-SARS-CoV-2 nucleocapsid Ig, memory impairment

## Abstract

Background: Post-COVID manifestation is defined as persistent symptoms or long-term complications beyond 4 weeks from disease onset. Fatigue and memory impairment are common post-COVID symptoms. We aimed to explore associations between the timeline and severity of post-COVID fatigue and anti-SARS-CoV-2 antibodies. Methods: Fatigue and memory impairment were assessed in a total of 101 post-COVID subjects using the Chalder fatigue scale (CFQ-11) and a visual analogue scale. Using the bimodal scoring system generated from CFQ-11, a score ≥4 was defined as severe fatigue. Serum anti-SARS-CoV-2 spike (anti-S-Ig) and nucleocapsid (anti-NC-Ig) antibodies were examined at two time points: 4–12 weeks after onset of symptoms, and beyond 12 weeks. Results: The serum level of anti-S-Ig was significantly higher in patients with non-severe fatigue compared to those with severe fatigue at 4–12 weeks (*p* = 0.006) and beyond 12 weeks (*p* = 0.016). The serum level of anti-NC-Ig remained high in patients with non-severe fatigue at both time points. In contrast, anti-NC-Ig decreased significantly in severe fatigue cases regardless of the elapsed time (4–12 weeks: *p* = 0.024; beyond 12 weeks: *p* = 0.005). The incidence of memory impairment was significantly correlated with lower anti-S-Ig levels (−0.359, *p* < 0.001). Conclusion: The systemic immune response reflected by antibodies to SARS-CoV-2 is strongly correlated with the severity of post-COVID fatigue.

## 1. Introduction

The SARS-CoV-2 pandemic has had a significant impact on national healthcare systems and economies worldwide, and has changed the lives of millions of people [1]. The illness COVID-19, caused by the SARS-CoV-2 virus, can manifest with a wide range of symptoms from asymptomatic or mild cases to moderate and even severe, life-threatening multisystemic disease [2]. Most initial studies focused mainly on the epidemiology, pathology and potential treatment options of the acute illness. As the number of recovered patients grows, managing those who continue to report adverse symptoms weeks or months after the cure of infection poses a new challenge.

Although initially thought to be primarily a respiratory infection, the SARS-CoV-2 virus affects multiple organ systems and requires a holistic diagnostic and treatment approach [3,4,5]. There seems to be a consensus for diagnostic criteria of COVID-19 worldwide [6,7]; however, the case definition of “post-COVID syndrome” or “long COVID” is more challenging. These patients report a vast number of diverse symptoms [8,9], making the interpretation of national statistics, the generalizability and comparison of various reports less straightforward. Several cross-sectional and cohort studies report that chronic fatigue is the most frequently reported symptom following recovery from acute COVID-19 [10]. Memory loss is also common among post-COVID patients [10]. Immunological factors may play a role in the pathomechanism of both [10], but the association with humoral immunity has not been extensively studied [10]. This paper aims to explore potential correlations between the serum level of SARS-CoV-2 Ig antibodies and the most commonly reported long COVID symptoms, such as fatigue and memory impairment.

## 2. Methods

### 2.1. Study Design and Population

This prospective single-centre cohort study was conducted at the Clinical Center of University of Pécs (UOP), Hungary with close collaboration of general practitioners (GPs) in the operation area of UOP.

Patients presenting with post-COVID symptoms at the GP office were scheduled for a post-COVID outpatient clinic (baseline visit) to assess their suitability (inclusion/exclusion criteria) for the study. In addition, patients were divided into two groups according to how much time had elapsed between the first symptoms of acute SARS-CoV-2 infection and the date of study enrolment (4–12 weeks vs. >12 weeks). This division was made according to the relevant National Institute of Care and Health Excellence (NICE) guideline [9]. After routine safety studies (BP, ECG) and blood sampling, patients completed the Chalder fatigue scale and two groups were formed based on the results (severe fatigue vs. non-severe fatigue, see Section 2.2). The severity of the acute illness (need for hospitalization vs. home care), symptoms associated with acute disease (0–4 weeks) and post COVID symptoms (>4 weeks) were also recorded. A post-COVID symptom was defined as any complaint that persisted after the index disease or occurred after week 4 and was not present before the time of infection. Laboratory testing and antibody determination were performed by another unit blinded to patients’ data. Inclusion criteria were as follows: (i) at least 30 days elapsed between the onset of symptoms and the date of the outpatient visit; (ii) all participants had at least one positive PCR test or a positive antigen test; (iii) symptomatic status at the time of outpatient appointment; and (iv) patients ≥ 18 years old. Exclusion criteria were as follows: (i) pre-existing malignant or active autoimmune disease; (ii) immunosuppressive therapy; (iii) acute coronary syndrome; (iv) vaccination against SARS-CoV-2; and (v) any condition that may interfere with the assessment of fatigue or cognitive state. Safety studies included 12-lead ECG, and routine laboratory tests were performed during baseline visit including white blood cell (WBC) count, red blood cell (RBC) count, haemoglobin level, serum level of creatinine and urea, serum level of thyroid-stimulating hormone (TSH) and high-sensitivity C-reactive protein (hsCRP), high-sensitive Troponin-T (hs-Tn-T), D-dimer, lactic acid dehydrogenase (LDH), and routine urine laboratory testing.

### 2.2. Fatigue and Symptom Severity Assessment

The Chalder fatigue scale (CFQ-11) was used to assess fatigue [11]. An English version of this validated score was translated by an official translation agency into Hungarian to be used in the study. All patients were asked to complete a questionnaire regarding their general condition in the last 3 days. With the use of the Likert scoring system, a score ranging from 0 to 3 was given. From this a global score could be constructed out of a total of 33. Scores for the subscales of physical (0–21) and psychological (0–12) fatigue were also calculated [12]. 

To create a case definition for severe fatigue, a bimodal scoring system was used ranging from 0 to 11 [12,13]. A score of 0 was given when the selected options were “less than usual” and “no more than usual”, while a score of 1 was given when “more than usual” and “much more than usual” were reported. Based on the bimodal scoring system, a score of 4 or more indicated caseness (or severe fatigue) [12,13].

The Post-COVID-19 Functional Status (PCFS) Scale was used to assess the general impact of post-COVID symptoms on the everyday life of each patient [14]. Only symptoms present in the 7 days prior to the baseline visit were considered in the assessment of symptoms. The severity of each symptom was rated using a visual analogue scale (VAS) ranging between 0 (no symptoms) to 10 (maximal symptom severity). Patients were asked to rate their three most disturbing symptoms and the sum of these formed the total VAS value indicating the overall severity of predominant symptoms. When a patient reported a decline in thinking abilities (including memory), it was defined as subjective cognitive impairment (SCI) [15], also known as subjective memory disorder. SCI is used as a catch-all term here.

### 2.3. Blood Sampling and Assay

Peripheral blood was collected and immediately centrifuged at 3500 r/min for 15 min. The supernatant was stored at −80 °C until analysis. Antibodies against SARS-CoV-2 were assessed in the peripheral blood by a fully automated Cobas e801 analyser (Roche Diagnostics). For the quantitative analysis of anti-SARS-CoV-2 spike protein antibodies the Elecsys^®^ Anti-SARS-CoV-2 S electrochemiluminescence immunoassay (Roche Diagnostics, Basel, Switzerland) was used. The assay uses a recombinant protein representing the receptor-binding domain (RBD) of the S antigen in a double-antigen sandwich assay format, which favours the detection of high-affinity antibodies against SARS-CoV-2. The qualitative detection of antibodies to the nucleocapsid protein was performed with the Elecsys^®^ Anti-SARS-CoV-2 electrochemiluminescence immunoassay (Roche Diagnostics, Basel, Switzerland). The assay uses a recombinant protein representing the nucleocapsid (N) antigen in a double-antigen sandwich assay format. The antigens within the reagents capture predominantly anti-SARS-CoV-2 IgG immunoglobulins (Igs), but also anti-SARS-CoV-2 IgA and IgM. These tests employ a sandwich reaction that includes both biotinylated and ruthenylated SARS-CoV-2 recombinant nucleocapsid and spike antigens incubated with the sample. The addition of streptavidin-coated microparticles allows the complex to be captured magnetically after binding to the solid phase through a biotin–streptavidin reaction. Electrochemiluminescence emission signals are interpolated to generate test results. Assay results were interpreted as follows: cut-off index <1.0, nonreactive/negative for antinucleocapsid antibodies; and cut-off index <0.8 U/mL for antispike antibodies.

### 2.4. Data Collection 

Demographic information and data about the index disease were collected from participants. Further information was obtained from patients’ electronic records including the clinically relevant data such as the start of the first symptoms, details of hospitalization, in- and outpatient care, antiviral medication, need for oxygen supplementation, regular medication, tobacco use, and premorbid cognitive state or dementia.

### 2.5. Ethical Considerations

The study was approved by the Hungarian Medical Research Council (IV/2505-3/2021/EKU). All procedures were performed in accordance with the ethical guidelines of the 1975 Declaration of Helsinki. Written informed consent was provided by all participants before enrolment in the present study.

### 2.6. Statistical Analysis

Data were evaluated using SPSS (version 11.5; IBM, Armonk, NY, USA). The Kolmogorov–Smirnov test was applied to check for normality. To analyse demographic and clinical factors, the chi-square test was used for categorical data while the Student’s *t*-test was applied to quantitative values. Non-normally distributed data were presented as median and interquartile range (25th–75th percentiles) and were compared with the use of Mann–Whitney test. Correlation analysis was performed by calculating Spearman’s correlation coefficient (rho). The best cut-off values of predictors were determined based on ROC analysis. A *p*-value < 0.05 was considered statistically significant.

## 3. Results

### 3.1. Participants Characteristics

**Figure 1:**
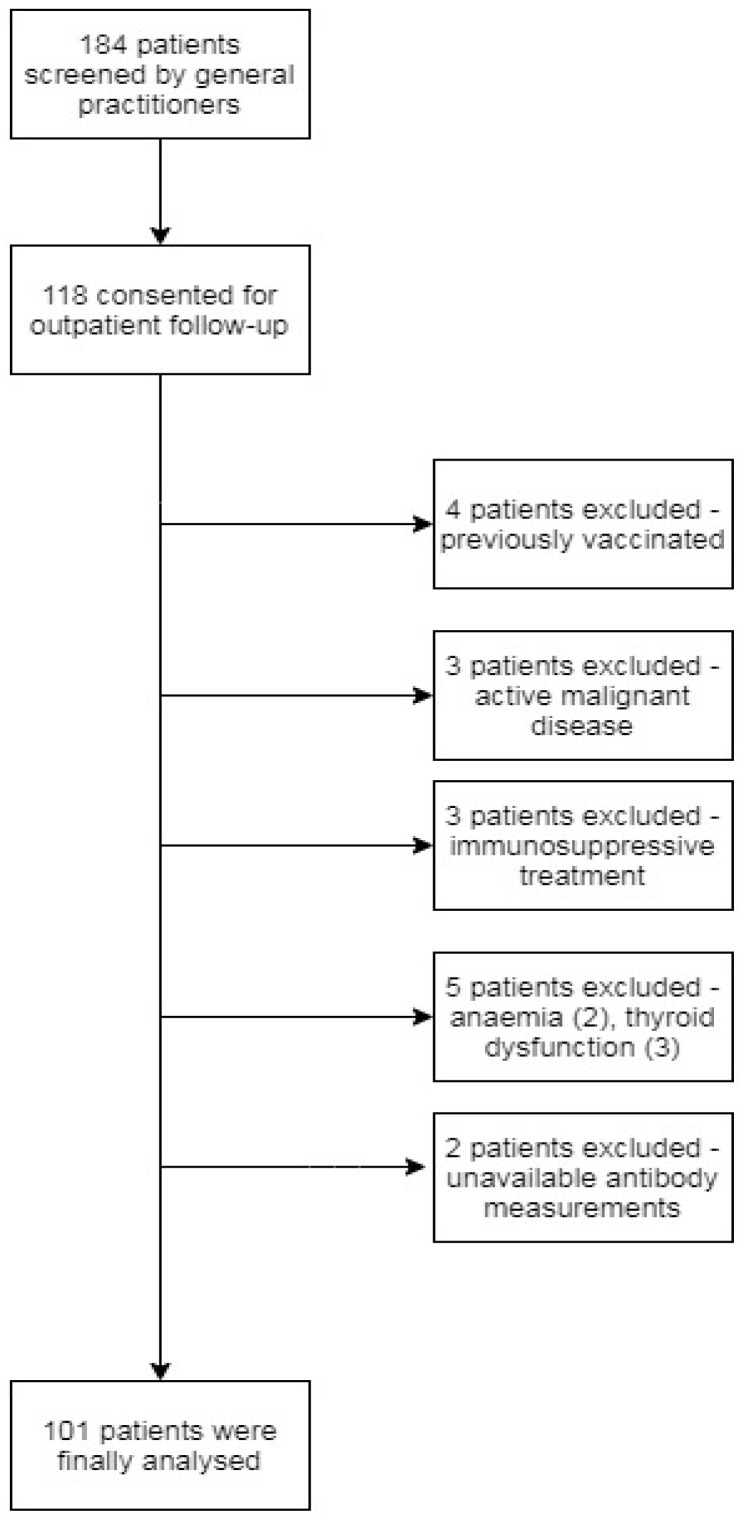
Patient selection for this prospective cohort study.

A total of 184 patients were eligible for participation in the study, of whom 118 consented for the outpatient baseline assessment and the collection of blood and urine samples. A total of 17 patients were excluded because they did not fulfil inclusion criteria at baseline visit (see Figure 1). 

Finally, data obtained from 101 patients (home care, *n* = 62, hospitalized, *n* = 39) were analysed. Table 1 shows the demographic and clinical characteristics of the cohort according to time elapsed from first symptoms to the date of the baseline visit. The mean (SD) age of enrolled participants was 50 years. All patients had symptoms associated with acute SARS-CoV-2 infection with a median number of symptoms of 6 (IQR: 4–7). At first presentation either in hospital or at the GP office, the most common symptoms related to acute SARS-CoV-2 infection were general weakness (79, 78.2%), fever (71, 70.3%), loss of smell (67, 66.3%), cough (62, 61.4%), muscle pain (56, 55.4%), headache (39, 38.6%), dyspnoe (34, 33.7%), diarrhoea (27, 26.7%), skin ache (25, 24.8%), joint pain (24, 23.8%), chest pain (20, 19.8%), sickness (19, 18.8%) and sore throat (16, 15.8%). The most common post-COVID symptoms mentioned by patients at the baseline visit were fatigue (68.3%), reduction in physical capacity (63.4%), palpitations (49.5%), sleep disturbances (34.7%) and subjective cognitive impairment (17.8%). The total number (median, IQR) of post-COVID symptoms reported by patients on baseline visit was 5 (2–8). Table 2 summarizes patient parameters on baseline divided into two groups based on fatigue scale assessment.

### 3.2. Serum Level of Anti-SARS-CoV-2 Antibodies in Patients with Different Phase of Long COVID Disease and Its Relation to Fatigue Status

**Figure 2:**
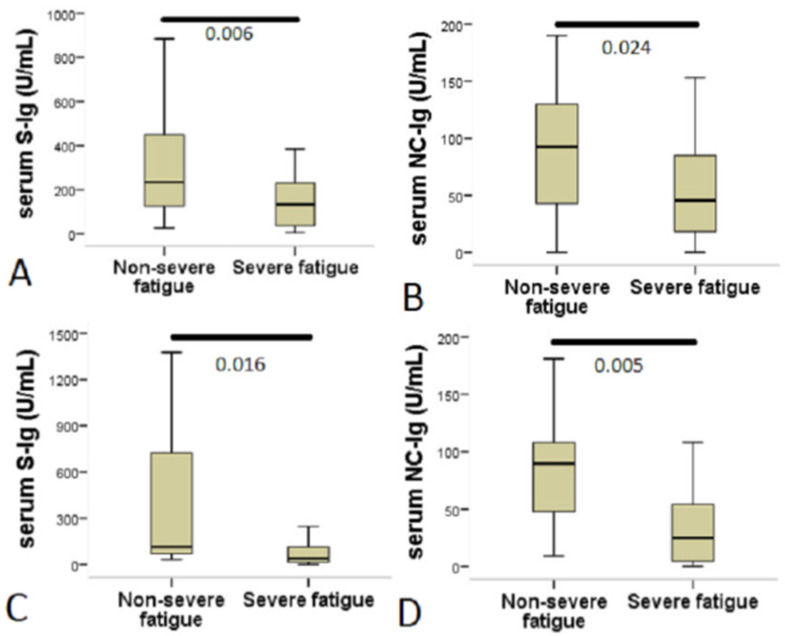
Patients with severe fatigue showed decreased serum anti-SARS-CoV-2 antibody levels. The figure shows a comparison of anti-SARS-CoV-2 antibody levels between patients with non-severe fatigue and those with severe fatigue. The Chalder fatigue scale (CFQ-11) was used to assess fatigue and to create a case definition for severe fatigue; a bimodal scoring system was used ranging from 0 to 11. A score of 4 or more indicated caseness (severe fatigue). (**A**,**C**) Serum anti-SARS-CoV-2 spike IgA + IgM + IgG levels (S-Ig). (**B**,**D**) Serum anti-SARS-CoV-2 nucleocapsid IgA + IgM + IgG levels (NC-Ig). Statistical analysis was performed using Mann–Whitney U test.

The level of anti-SARS-CoV-2 spike Ig (anti-S-Ig) in the serum of post-COVID patients was higher in symptomatic patients at 4–12 weeks compared to post-COVID patients beyond 12 weeks (Table 1). The serum level of both S-Ig and anti-SARS-CoV-2 nucleocapsid Ig (anti-NC-Ig) were elevated in patients with non-severe fatigue compared to those with severe fatigue (Table 2). The level of serum anti-S-Ig was significantly higher in patients with non-severe fatigue compared to those with severe fatigue in symptomatic patients at 4–12 weeks (non-severe fatigue: 235 U/mL, IQR: 125–450 vs. severe fatigue: 132.5 U/mL, IQR: 38–230, *p* = 0.006) as well as in post-COVID patients beyond 12 weeks (non-severe fatigue: 114 U/mL, IQR: 70–723 vs. severe fatigue: 38 U/mL, IQR: 16–113, *p* = 0.016). This was more pronounced in the earlier stage after the index event (Figure 2A,C). 

In contrast, the serum level of anti-NC-Ig remained high in patients with non-severe fatigue in both examined groups, whereas in patients with severe fatigue the anti-NC-Ig level was significantly lower regardless of the elapsed time from index disease (symptomatic patients at 4–12 weeks: non-severe fatigue: 92.5 U/mL, IQR: 43–130 vs. severe fatigue: 45.4 U/mL, IQR: 18–85, *p* = 0.024; post-COVID patients beyond 12 weeks: non-severe fatigue: 90 U/mL, IQR: 48–108 vs. severe fatigue: 24.8 U/mL, IQR: 4–54, *p* = 0.005) (Figure 2B,D). The area under the curve (AUC) for serum anti-NC-Ig level as a predictor of severe fatigue in post-COVID patients beyond 12 weeks was 0.79 (95% CI = 0.632–0.947; *p* < 0.006). Based on the best cut-off value by a ROC analysis, serum anti-NC-Ig level <43.8 U/mL predicted severe fatigue status in this group of patients with a sensitivity of 83.3% and a specificity of 71.4%.

### 3.3. Relationship between Demography, Laboratory and Clinical Features at Baseline and Anti-SARS-CoV-2 Antibodies

Serum levels of anti-S-Ig were significantly lower in patients <50 years than in those aged 50 years and above (66.1 U/mL, IQR: 25–171 vs. 189 U/mL, IQR: 124–450, *p* < 0.001). A correlation was also observed for anti-NC-Ig (41.8 U/mL, IQR: 13–66 vs. 84.9 U/mL, IQR: 25–108, *p* = 0.028). Significantly higher serum levels of anti-S-Ig were also found in the hospitalized group compared to the non-hospitalized group (67.6 U/mL, IQR: 28–144 vs. 246 U/mL, IQR: 151–473, *p* < 0.001). In contrast, anti-NC-Ig showed no correlation with hospitalization. 

At baseline visit, the incidence of SCI was significantly correlated with lower anti-S-Ig levels (144 U/mL, IQR: 62–324 vs. 26.2 U/mL, IQR: 12–101, *p* < 0.001). In addition, patients with SCI had a higher CFQ-11 score (20, IQR: 18–22 vs. 15, IQR: 11–19, *p* < 0.001), were younger (45.7 ± 10 vs. 50.9 ± 12, *p* = 0.041) and had more symptoms at baseline (7 ± 3 vs. 5 ± 2, *p* = 0.006) than those without SCI at baseline visit. Sleep disorders (56% vs. 30%, *p* = 0.04), “brain fog” (27.8% vs. 1.2%, *p* > 0.001) and depression (27.8% vs. 8.4%, *p* = 0.021) were also more common among patients with SCI than those without memory problems. Other variables associated with anti-SARS-CoV-2 antibodies on cross-sectional analysis are depicted in Table 3. 

Variables associated with severe fatigue status were entered into a binary logistic regression model, where the serum level of anti-S-Ig and total number of symptoms at baseline were independently associated with severe fatigue status at baseline, whereas age, level of anti-NC-Ig, total VAS score and total number of symptoms on admission were not (Table 4). Next, a separate statistical analysis was run with median values of anti-S-Ig and anti-NC-Ig as an outcome of interest. Based on binary logistic regression analysis, the total value of CFQ-11 score proved to be an independent predictor of median anti-NC-Ig level, while the total value of CFQ-11 scale and need for hospitalization were independently associated with the median anti-S-Ig level.

## 4. Discussion

In the present study, we examined the association between SARS-CoV-2 antibody levels and data from patients with post-COVID symptoms. Patients were categorized into two groups based on the time elapsed between the first symptoms of acute SARS-CoV-2 infection and the date of study enrolment (4–12 weeks vs. >12 weeks). The most common post-COVID symptoms reported by patients were fatigue, reduction in physical capacity, palpitations, sleep disturbances and subjective cognitive impairment. We found that female sex was more prevalent in both the entire patient population and in those with symptoms of severe fatigue. Similarly, the incidence of post-COVID features has recently been reported to be significantly higher among women [16]. Nevertheless, the literature is inconsistent regarding the female dominance of post-COVID syndrome (PCS). In a large cohort study, sex did not behave as an independent predictor of PCS [17], but similar to our results, female sex was significantly more common in patients with PCS in another study [18]. In accordance with our findings, female sex was more prevalent in the severely fatigued group [18]. Independently of PCS, previous reports have indicated a marked female dominance in chronic fatigue syndrome, which may also suggest a gender-specific pathophysiological process [19].

Importantly, the most frequent symptom reported in our cohort was fatigue (68%) and both the mean value of total CFQ-11 score and the time course of fatigue in our participants were similar to those observed by other authors [18]. Therefore, our study population was suitable for exploring the relationship between antibody response after SARS-CoV-2 and post-COVID fatigue. Anti-SARS-CoV-2 antibody titres were significantly lower in patients under 50 years of age in our cohort. Our findings are in agreement with other studies showing that older age is associated with higher antibody titres in the plasma [20,21,22]. Elderly patients also had significantly higher titres of neutralizing antibodies after COVID-19 and higher serum level of CRP at the time of hospital discharge [23]. We found a significantly higher anti-S-Ig titre in patients requiring hospitalization, while the level of anti-NC-Ig showed no correlation with hospitalization. This finding is also in line with numerous studies examining the relationship between symptomatic status and antibody response to COVID-19 infection [22,24,25,26,27]. Importantly, our cohort included only symptomatic patients, thus we could only compare antibody levels in patients with mild to moderate symptoms. The difference in levels of anti-S-and anti-NC-Ig shown in the hospitalized group in our cohort is intriguing. A previous quantitative analysis of the binding antibody response to the spike and nucleocapsid proteins found a heterogeneous response, and the authors concluded that antibody titres against NC protein may suggest prior exposure to SARS-CoV-2 or related viruses, but do not necessarily provide evidence for the presence of neutralizing antibodies [28]. In our study, we found that the anti-S-Ig titre was higher in patients recruited between 4 and 12 weeks after the onset of symptoms compared to those enrolled beyond 12 weeks, while the titre of anti-NC-Ig did not follow this pattern. Our findings are consistent with previous studies showing a decrease in antibody titres over time [22,24,25,29]. However, antibody titres were preserved during follow-up, irrespective of the severity of symptoms in another study, and patients still had detectable Ig levels even beyond 75 days [26]. In our study, the elapsed time between symptom onset and study enrolment was not an independent predictor of either anti-S-Ig or anti-NC-Ig levels. A previous study demonstrated that immunoglobulins and neutralizing antibodies against SARS-CoV-1 may persist for 2–3 years in recovering patients despite a declining titre [30]. Overall, it is difficult to extrapolate the results from previous studies due to their different sensitivity and specificity features. Longitudinal studies are needed, as the pattern of decrease in antibody titre may be influenced by several factors. 

The key finding of our study is that significantly lower anti-S-Ig and anti-NC-Ig levels were found in patients with severe fatigue, regardless of the time elapsed from the onset of the disease. Moreover, the value of the CFQ-11 scale was an independent predictor of the NC-Ig titre measured at baseline. It has been proposed that the pro-inflammatory environment during late convalescence (≥90 days after symptom onset) could be important in maintaining long-term SARS-CoV-2-specific neutralising antibody levels [31]. We found that both monocyte and leukocyte counts measured at baseline showed a significant positive correlation with anti-S-Ig titre, while the level of anti-NC-Ig showed a similar association with eosinophil count, suggesting a possible link between antibody level and immune cells. The exact cause of post-COVID fatigue is still unknown, but in many features it shows similarities to other chronic fatigue syndromes (CFSs) [32]. A study examining the relationship between immune function and CFS after infectious mononucleosis found that a subset of patients had undetectable Epstein–Barr nuclear antigen (EBNA) Ig titres and diminished immune response against EBV [33]. Our theoretical consideration is that chronic fatigue may be caused by chronic inflammation, and the lower level of antibodies in patients with severe fatigue indicate an unsatisfactory immune response against the viral pathogen. Based on analogy with other similar syndromes, there are several possible mechanisms that might explain this theory. Presumably, the immune system of some patients is poorly activated, resulting in a decreased production of immunoglobulins, slower viral clearance from infected cells, and thus the maintenance of a prolonged inflammatory state which manifests in chronic fatigue. This can be seen after Q fever, where elevated levels of IFN-gamma, IL-1 and IL-6 may underlie chronic fatigue. Q fever fatigue syndrome (QFS) patients show signs of altered immunity and inflammatory profile compared to asymptomatic Q fever seropositive controls [34]. The expression of mitochondrial-derived peptide (MDP)-coding genes MT-RNR1 (MOTS-c) and MT-RNR2 (humanin) are decreased in CFS, QFS and to a lesser extent in Q fever seropositive controls, resulting in a decreased production of humanin. These peptides might be important in the pathophysiology of both QFS and CFS [35,36]. Another mechanism which could contribute to the alterations in antibody response against COVID-19 is that the different spike or nucleocapsid variations affect the immune mechanism in a similar way to EBV infection regarding the EBNA variations [33]. A further possibility is that COVID-19-associated changes in the intestinal microbiome could be a possible consequence. In case of Q fever, it has been shown that the *Bacteroidetes* and *Firmicutes* abundance are reduced in chronic fatigue syndrome compared to healthy individuals [37]. In COVID-19, an increase in *Collinsella* spp. has been observed. This bacterium has an important role in the maintenance of chronic inflammation [38]. Given the paucity of evidence available, further studies are needed to clarify the exact mechanism of fatigue after COVID-19 infection. 

At baseline, the incidence of SCI was significantly correlated with lower anti-S-Ig levels. In the acute phase of COVID-19, the systemic levels of cytokines are elevated [39]. These cytokines can cross the blood–brain barrier, activate microglia and release IL-1β targeting the postsynaptic receptors of hippocampal neurons [40]. This renders the hippocampus vulnerable to IL-1β, which has been shown to disrupt memory [41]. The other mechanism that is likely to play a role in memory impairment during COVID-19 is the decrease of ACE2-mediated brain-derived neurotrophic factor (BDNF) activity [42]. ACE2 regulates normal brain function by stimulating BDNF activity [43]. Low BDNF levels were associated with cognitive impairment in a human study [44]. These pathologies might persist in the post-COVID period, but their contributions to SCI have not been investigated in our study. Consequently, the observed association between low anti-S-Ig levels and SCI requires further investigation. A recent prospective study reporting a beneficial effect of vaccination on physical and mental composite score supports our observed association between antibody levels and post-COVID symptoms such as SCI [45]. The authors assessed 44 vaccinated participants at 1 month after vaccination and compared them with 22 matched unvaccinated participants, and found that those who had received a vaccine experienced an increase in symptom resolution (23.2% vaccinated vs. 15.4% unvaccinated). The lower level of serum antibodies in patients with severe fatigue may indicate insufficient immune response to the index infection, resulting in persisting symptoms. Considering these findings, we speculate that the pronounced immune response induced by the COVID-19 vaccine may promote and activate the entire immune system to eliminate the remaining viral antigens and lead to the improvement of symptoms. 

Our study has some limitations. A relatively small sample size was used, and the single antibody measurement limits our ability to evaluate antibody response in a longitudinal setting. Furthermore, an official translation of the CFQ-11 scale per se does not guarantee cross-cultural adaptation and validity. Although the original English version of the CFQ-11 scale is validated, fatigue is a fairly subjective phenomenon and is difficult to measure. Additionally, no imaging studies were performed at baseline to examine structural abnormalities in patients with post-COVID symptoms. Only humoral immunity was studied in our cohort; we have no information on cellular immunity, although this is an important factor in immunity against SARS-CoV-2. Finally, memory impairment was examined based on self-reported patient complains, and validated neuro-cognitive tests were not included.

## 5. Conclusions

The serum level of anti-S-Ig was significantly higher in patients with non-severe fatigue compared to those with severe fatigue both at 4–12 weeks and beyond 12 weeks post-COVID. Similarly, the serum level of anti-NC-Ig remained high in patients with non-severe fatigue at both time points, whereas in patients with severe fatigue, anti-NC-Ig levels significantly decreased regardless of the elapsed time. In addition, the incidence of self-reported memory impairment was correlated with lower anti-S-Ig levels at baseline. Although the cellular immune response was not investigated here, based on these findings, the strength of systemic immune response reflected by anti-SARS-CoV-2 antibody titres may have an impact on both the severity of post-COVID fatigue and memory impairment.

## Figures and Tables

**Table 1:** Characteristics of the eligible patients with post-COVID symptoms according to time elapsed from first symptoms to date of baseline visit.

	Total Population (*N* = 101)	4–12 Weeks (*N* = 68)	>12 Weeks (*N* = 33)	*p*-Value
Age (mean ± SD)	50 ± 12	51.1 ± 11.5	47.7 ± 12.6	0.118
Female (N (%))	60 (59.4)	41 (60.3)	21 (63.6)	0.746
BMI (mean ± SD)	27.8 ± 6.4	27.5 ± 5	28.3 ± 9	0.476
Symptom onset to baseline visit (days; mean ± SD)	70.1 ± 30	52.7 ± 18	106 ± 13	<0.001
Hospitalization (N (%))	39 (38.6)	34 (50)	5 (15.2)	0.001
PCFS (median (IQR))	2 (1–2)	2 (1–2)	1 (1–2)	0.766
Total value of post-COVID symptoms on a VAS scale * (median (IQR))	16 (9–19)	16 (9–19)	16 (10–21)	0.204
Total CFQ-11 score (mean ± SD) (Likert scoring)	15.7 ± 5.9	15.8 ± 5.5	15.6 ± 6.7	0.951
Physical fatigue (mean ± SD) (CFQ-11 items 1–7)	11.7 ± 4.2	11.7 ± 3.8	11.8 ± 4.9	0.668
Psychological fatigue (mean ± SD) (CFQ-11 items 8–11)	4.1 ± 2.6	4.2 ± 2.5	3.9 ± 2.8	0.619
Total CFQ-11 score (mean ± SD) (bimodal scoring)	4.9 ± 3.2	4.9 ± 3	5 ± 3	0.773
Anti-SARS-CoV-2 S-Ig (U/mL; median (IQR))	125 (40–289)	169.5 (58–308)	70.8 (31–131)	0.037
Anti-SARS-CoV-2 NC-Ig (U/mL; median (IQR))	53.9 (18–105)	54.7 (21–107)	41.8 (9–97)	0.201

Data are presented as mean ± SD or median (interquartile range) as appropriate. Proportions are expressed as numbers, with percentages given in brackets. The significance of inter-group differences were assessed using chi-square test or Fisher exact test for categorical data, as well as Student’s t-test or Mann–Whitney U test for continuous variables. Abbreviations: N, number; BMI, body mass index; PCFS, Post-COVID Functional Status Scale; VAS, visual analogue scale; CFQ-11, Chalder fatigue scale; COVID, coronavirus disease; SARS, severe acute respiratory syndrome; S-Ig, spike immunoglobulin; NC-Ig, nucleocapsid immunoglobulin. * The sum of the VAS score given for the three most severe symptoms.

**Table 2:** Characteristics of study population by fatigue case status at baseline visit.

	Non-Severe Fatigue (*N* = 38)	Severe Fatigue (*N* = 63)	*p*-Value
Age (mean ± SD)	52.2 ± 13	48.7 ± 11	0.064
Female (N (%))	18 (47.4)	44 (69.8)	0.025 *
BMI (mean ± SD)	28.5 ± 8	27.3 ± 5	0.468
Symptom onset to baseline (day; mean ± SD)	71.2 ± 26	69 ± 32	0.641
Hospitalization (N (%))	16 (42.1)	23 (36.5)	0.576
Total number of comorbidities (median (IQR))	1 (0–2)	1 (0–1)	0.658
PCFS (median, IQR)	1 (0–1)	2 (1–2)	<0.001
Length of hospitalization (day, mean ± SD)	3.2 ± 4	2.3 ± 3.5	0.370
CTss (median (IQR))	9 (6–11)	11 (7–13)	0.217
O_2_ supplementation (N (%))	8 (21)	14 (22)	0.890
Antiviral medication (N (%))	15 (39.5)	19 (30.2)	0.337
CRP (mg/L; median (IQR))	52.9 (14–81)	29.5 (12–79)	0.630
High-sensitivity Troponin-T (ng/L; median (IQR))	10.4 (4–13)	6.6 (5–9)	0.201
NLR (median (IQR))	3.2 (2–6)	4.3 (2–6)	0.476
IL-6 (pg/mL; median (IQR))	29 (12–36)	28 (20–48)	0.580
D-dimer (µg/L; median (IQR))	738 (573–1009)	740 (412–1159)	0.730
Ferritin (µg/L; median (IQR))	552 (354–899)	569 (462–881)	0.695
Total CFQ-11 score (mean ± SD) (Likert Scoring)	9.9 ± 3.7	19.2 ± 3.7	<0.001
Physical fatigue (mean ± SD) (CFQ-11 items 1–7)	7.3 ± 2.6	14.3 ± 2.6	<0.001
Psychological fatigue (mean ± SD) (CFQ-11 items 8–11)	2.6 ± 1.9	5 ± 2.6	<0.001
Total CFQ-11 score (mean ± SD) (bimodal scoring)	1.5 ± 1.4	7 ± 1.9	<0.001
Number of post-COVID symptoms (median (IQR))	2 (1–3)	7 (5–10)	<0.001
Total value of post-COVID symptoms on a VAS scale * (median (IQR))	6 (0–14)	18 (15–21)	<0.001
Anti-SARS-CoV-2 S-Ig (U/mL; median (IQR))	211 (103–473)	72.7 (25–201)	<0.001
Anti-SARS-CoV-2 NC-Ig (U/mL; median (IQR))	91.8 (46–125)	34.5 (10–66)	<0.001

The categorical variables are presented as frequency (%) and the continuous variables are presented as mean ± standard deviation (SD) or median with interquartile range (IQR). The inter-group differences were assessed using chi-square test, Fisher exact test, Student’s *t*-test or Mann–Whitney U test as appropriate in order to compare differences in those without non-severe fatigue and those with severe fatigue as per the CFQ-11 “caseness” definition for severe fatigue. Abbreviations: N, number; BMI, body mass index; PCFS, Post-COVID Functional Status Scale; CTss, computer tomography severity score; CRP, C-reactive protein; NLR, neutrophile—lymphocyte ratio; IL-6, interleukin-6; VAS, visual analogue scale; CFQ-11, Chalder fatigue scale; COVID, coronavirus disease; SARS, severe acute respiratory syndrome; S-Ig, spike immunoglobulin; NC-Ig, nucleocapsid immunoglobulin. * The sum of the VAS score is given for the three most severe symptoms.

**Table 3:** Cross-sectional analysis among SARS-CoV-2 antibodies and variables at baseline.

Variables	S-Ig	NC-Ig
Total number of symptoms at baseline	−0.229 *	−0.237 *
Total number of comorbidities	0.225 *	0.108
Body mass index	0.249 *	0.175
Length of hospitalization	0.519 **	0.230
Admission GOT **	0.233	−0.476 *
Admission GPT **	0.159	−0.334 *
Admission platelet **	0.344 *	0.148
Admission hs-Tn-T **	−0.430 *	0.065
Baseline lymphocyte count	0.251 *	0.133
Baseline monocyte count	0.337 **	0.069
Baseline eosinophile count	0.147	0.233 *
Total score on VAS *	−0.162	−0.292 *
Baseline GOT	0.245 *	0.062
Baseline GPT	0.318 **	0.006
Baseline GGT	0.287 *	−0.013
Baseline hs-Tn-T	0.295 *	0.148
Baseline LDH	0.327 **	0.124

Values are Spearman correlation coefficients (rho). * *p* < 0.05, ** *p* < 0.001. Abbreviations: GOT, glutamic oxaloacetic transaminase; GPT, glutamic pyruvic transaminase; GGT, gamma-glutamyl transferase; hs-Tn-T, high-sensitive Troponin-T; LDH, lactate dehydrogenase. * The sum of the VAS score is given for the three most severe symptoms. ** For those patients who were hospitalized during acute SARS-CoV-2 infection.

**Table 4:** Results of the binary logistic regression analysis examining associations between anti-SARS-CoV-2 antibody levels, severe fatigue status and demographic/clinical variables.

		**Value of NC-Ig (U/mL, Median as the Cutoff) ^§^**		
Variables	B	Odds ratio	95% CI	*p*-Value
Symptom onset to baseline (days)	0.993	0.660	0.976–1.010	0.417
Total CFQ-11	0.883	9.037	0.814–0.958	0.003
Age	1.031	2.512	0.993–1.071	0.113
Need for hospitalization	0.908	0.030	0.308–2.676	0.862
		Value of S-Ig (U/mL, Median as the Cutoff) ^§^		
Variables	B	Odds ratio	95% CI	*p*-value
Symptom onset to baseline (days)	0.985	2.337	0.966–1.004	0.126
Total CFQ-11	0.905	4.968	0.829–0.988	0.026
Age	1.045	3.929	1.000–1.091	0.047
Need for hospitalization	0.169	8.772	0.052–0.548	0.003
		Severe Fatigue Status		
Variables	B	Odds ratio	95% CI	*p*-value
Age	0.950	1.811	0.882–1.024	0.178
S-Ig	0.997	4.688	0.994–1.000	0.030
NC-Ig	0.993	1.105	0.980–1.006	0.293
Total VAS *	1.096	1.078	0.922–1.303	0.299
Total number of symptoms at baseline	2.021	6.917	1.196–3.413	0.009
Sex	0.885	0.024	0.190–4.117	0.877
Total number of symptoms at baseline	1.159	0.801	0.839–1.601	0.371

^§^ In these binary logistic regression models, serum antibody levels were converted to a binary dependent variable, based on the median value of the sample (0: ≤median, 1: >median). Abbreviations: NC-Ig, nucleocapsid immunoglobulin A + M + G); S-Ig, spike immunoglobulin IgA + IgM + IgG; VAS, visual analogue scale; CFQ-11, Chalder fatigue scale; COVID, coronavirus disease; SARS, severe acute respiratory syndrome. * The sum of the VAS (visual analogue scale) score given for the three most severe symptoms.

## Data Availability

All relevant data are within the manuscript.
